# Label-Free Assessment of Neuronal Activity Using Raman Micro-Spectroscopy

**DOI:** 10.3390/molecules29133174

**Published:** 2024-07-03

**Authors:** Yuka Akagi, Aya Norimoto, Teruhisa Kawamura, Yasuyuki S. Kida

**Affiliations:** 1 Cellular and Molecular Biotechnology Research Institute, National Institute of Advanced Industrial Science and Technology (AIST), Central 5, 1-1-1 Higashi, Tsukuba 305-8565, Ibaraki, Japan; y-akagi@aist.go.jp (Y.A.); aya.norimoto@aist.go.jp (A.N.); 2 Department of Biomedical Sciences, College of Life Sciences, Ritsumeikan University, 1-1-1 Noji-Higashi, Kusatsu 525-8577, Shiga, Japan; kawater@fc.ritsumei.ac.jp; 3 School of Integrative & Global Majors, University of Tsukuba, 1-1-1 Tennoudai, Tsukuba 305-8572, Ibaraki, Japan

**Keywords:** Raman spectroscopy, neuronal activity, induced pluripotent stem cells, non-label evaluation

## Abstract

Given the pivotal role of neuronal populations in various biological processes, assessing their collective output is crucial for understanding the nervous system’s complex functions. Building on our prior development of a spiral scanning mechanism for the rapid acquisition of Raman spectra from single cells and incorporating machine learning for label-free evaluation of cell states, we investigated whether the Paint Raman Express Spectroscopy System (PRESS) can assess neuronal activities. We tested this hypothesis by examining the chemical responses of glutamatergic neurons as individual neurons and autonomic neuron ganglia as neuronal populations derived from human-induced pluripotent stem cells. The PRESS successfully acquired Raman spectra from both individual neurons and ganglia within a few seconds, achieving a signal-to-noise ratio sufficient for detailed analysis. To evaluate the ligand responsiveness of the induced neurons and ganglia, the Raman spectra were subjected to principal component and partial least squares discriminant analyses. The PRESS detected neuronal activity in response to glutamate and nicotine, which were absent in the absence of calcium. Additionally, the PRESS induced dose-dependent neuronal activity changes. These findings underscore the capability of the PRESS to assess individual neuronal activity and elucidate neuronal population dynamics and pharmacological responses, heralding new opportunities for drug discovery and regenerative medicine advancement.

## 1. Introduction 

Neurons, which are the primary units of the brain, spinal cord, and peripheral nervous system, are pivotal for receiving, processing, and transmitting information. Within the brain, structures known as nuclei and regions of gray matter comprise neuronal populations that are essential for processing information, sensory transmission, motor control, and facilitating cognitive and memory functions through electrical and chemical signaling. In the peripheral nervous system, neurons form ganglia, which play crucial roles in sensory transmission, motor control, and the regulation of organ functions via their interactions. Evaluating neuronal output at the population level is critical for gaining new insights into and analyzing the intricate operations of the nervous system, with implications across medicine, biology, and pharmacology, including the study of neurodegenerative diseases, treatment development, and neuronal circuit reconstruction.

Raman spectroscopy, named after its discovery by Raman and Krishnan in 1928, serves as an analytical tool to determine the molecular structure and chemical composition of materials [1]. This technique involves illuminating a sample with a laser and analyzing the light scattered from it. The frequency shifts in the scattered light, known as Raman shifts, result from interactions with the molecular vibrations of the sample, providing a distinct spectral signature that enables the nondestructive identification of molecular contents and insights into structural configurations. The ability of Raman spectroscopy to deliver detailed molecular information without altering or destroying the sample renders it invaluable for various biological applications, such as cell and tissue analysis [2,3,4], disease diagnostics [5,6,7], and biochemical process studies [8].

To enhance the application of this technique in biological research and assessments, including medical diagnostics and food science quality control, we developed the Paint Raman Express Spectroscopy System (PRESS) [9]. This system is tailored for the rapid and accurate acquisition of Raman spectra from whole cells by employing a spiral-scanning mechanism to direct a laser beam across a specific circular area. During this laser irradiation, the shutter remains open. This setup facilitates the collection of an integrated Raman spectrum from the illuminated region within a few seconds. For example, when analyzing a single cell within a 10 µm diameter region, with a scanning velocity of 1 mm ms^−1^ and an exposure duration of 3 s, the spiral scan is executed 34 times. As the shutter is open during this time, scattered light from the area is collected, and a spectrum with a suitable signal-to-noise ratio for analysis is obtained. This method allows the PRESS to offer a detailed overview of the molecular environment within a scanned area, such as individual cells, providing insights into the chemical and structural makeup at the cellular level. Furthermore, the integration of machine learning with spectral data facilitates the precise identification of cell types and states with an accuracy exceeding 90%. In this study, we hypothesized that the expansive measurement capability and rapid functionality of the PRESS are well suited for evaluating both individual neuronal activity and the collective output of the neuronal population.

To test this hypothesis, we examined neuronal activity using individual glutamatergic neurons, which constitute the majority of the brain, and autonomic neurons, which form the ganglia in the peripheral nervous system. The chemical responses were observed using the PRESS. Subsequent analysis of Raman spectra using deep learning techniques revealed that the PRESS can detect changes in the activity of individual neurons as well as neuronal populations. These findings suggest that the PRESS may provide insights into neuronal dynamics and pharmacological effects, thereby opening new avenues for applications in drug discovery and regenerative medicine.

## 2. Results

### 2.1. Evaluation of Individual Neuronal Activities Using the PRESS

**Figure 1:**
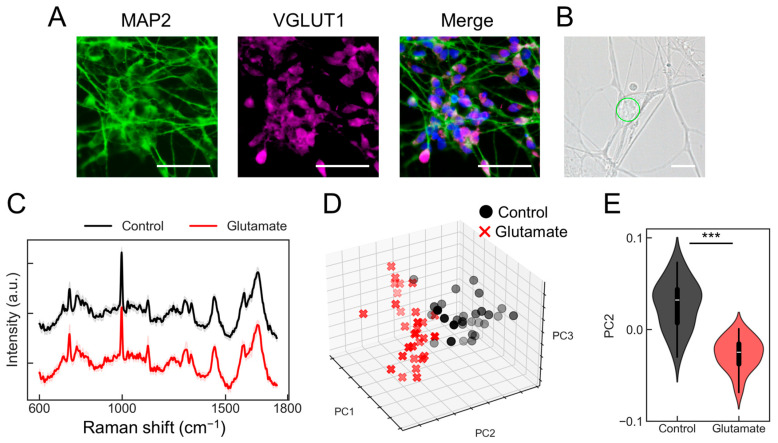
Evaluation of individual neurons activity using the Paint Raman Express Spectroscopy System (PRESS). (**A**) Immunofluorescent staining shows induced glutamatergic neurons at 20 days post-induction, pan-neuronal marker MAP2, and glutamatergic neuron marker vesicular glutamate transporter 1 (VGLUT1). The merge diagram shows blue: nucleus (Hoechst), green: mature neuron (MAP2), and magenta: glutamatergic neuron (VGLUT1). The scale bar represents 100 µm. (**B**) A bright field microscopy image displaying the morphological features of induced glutamatergic neurons on day 20 post-induction. The scale bar represents 10 µm. The area subjected to measurement is demarcated by a green line. (**C**) Average Raman spectra obtained from glutamatergic neurons stimulated with 10 mM glutamate (red) or control buffer (black). Standard deviations (SDs) are shown by shaded areas. These data are the result of measurements taken from 30 neurons (*n* = 30). (**D**) A three-dimensional (3D) plot representing the distribution of individual glutamatergic neurons along the PC1, PC2, and PC3 axes from the PCA (control samples: black circles; glutamate-stimulated samples: red crosses [x]). (**E**) Violin plots comparing values for samples treated with glutamate or the control against the PC2 axes (*n* = 30; data were analyzed using the Student’s *t*-test; ***: *p* < 0.001 vs. the control).

First, we explored whether the PRESS could be utilized to assess changes in individual neuronal activity. In this study, glutamatergic neurons derived from human-induced pluripotent stem cells (hiPSCs) were used. Several studies have demonstrated the importance of SMAD signaling in neuronal induction [10,11]. Therefore, neuronal differentiation was promoted using a method called “dual SMAD inhibition,” which employs dorsomorphin, an inhibitor of bone morphogenetic protein (BMP) signaling (SMAD1/5/8), and SB431542, an inhibitor of TGF/activin/nodal signaling (SMAD2/3) (Figure S1A). The induced neurons expressed markers of mature neurons, such as MAP2, and markers of glutamatergic neurons, such as vesicular glutamate transporter 1 (VGLUT1) (Figure 1A). Additionally, calcium imaging using the calcium indicator Fluo8 showed that these neurons responded to glutamate (Figure S1B). The responsiveness of the induced individual glutamatergic neurons to glutamate was tested to determine whether it could be detected using the PRESS. We configured the laser to perform a spiral scan over a 10 µm diameter circle during a 3 s exposure period, ensuring that a single spectrum was obtained from each neuron (Figure 1B). Thirty cells were selected immediately after exposure to a control solvent or glutamate, and Raman spectra were acquired from each. After the pre-processing steps, such as noise reduction and baseline correction, the spectral data showed similar peak patterns. Figure 1C shows the spectral data for the control (black lines) and glutamate (red lines) samples. Principal component analysis (PCA) of the acquired spectral data revealed distinct distributions between the control and glutamate-stimulated groups (Figure 1D and Figure S1C, control: black plot, glutamate: red plot). The contributions from each principal component (PC1, PC2, and PC3) were 17.9%, 3.9%, and 3.3%, respectively, with significant differences observed particularly along PC2 (*p* = 6.93 × 10^−13^; *n* = 30) (Figure 1E). Furthermore, evaluation of the classification accuracy using partial least squares discriminant analysis (PLS-DA) yielded an area under the curve (AUC) of 0.99, a root mean square error (RMSE) of 0.03, a sensitivity of 1.00, and a specificity of 0.98 (Figure S1D). These results demonstrate that the PRESS can be used to evaluate activity changes in individual neurons.

### 2.2. Acquisition of Raman Spectra of the Autonomic Neuron Ganglia 

**Figure 2:**
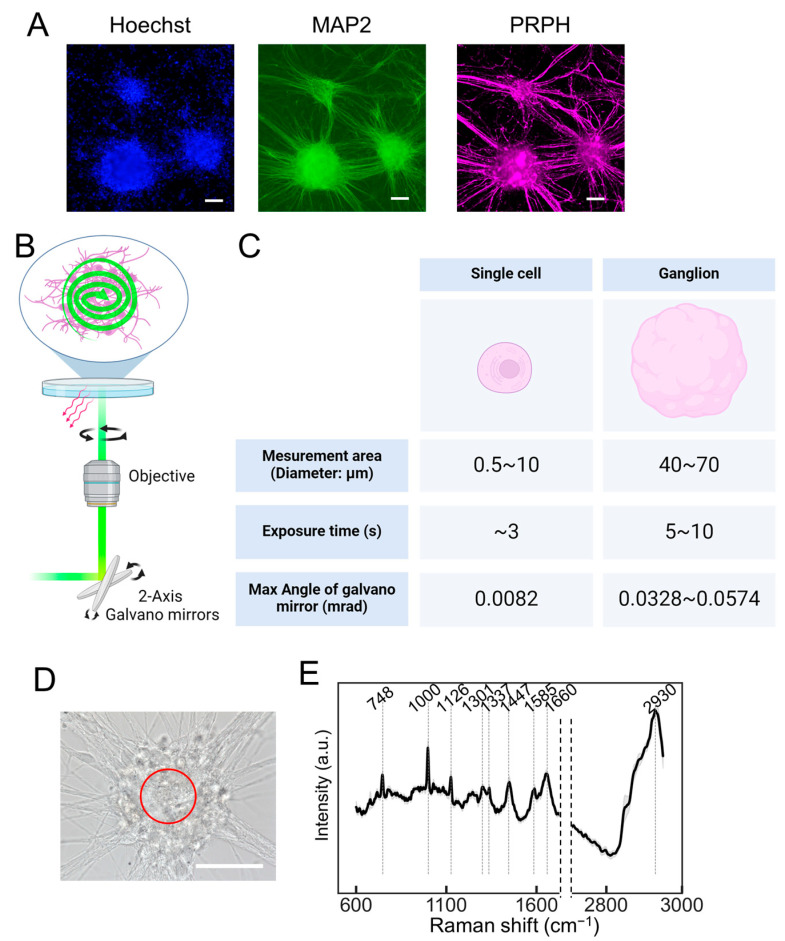
The Raman spectroscopy of autonomic neurons (ANs) ganglia utilizing the PRESS. (**A**) Immunofluorescent staining showing induced neurons at 40 days post-induction, highlighting the nucleus with Hoechst, the pan-neuronal marker MAP2, and the peripheral neuron marker peripherin (PRPH). The scale bar represents 100 µm. (**B**) A schematic representation of the PRESS, illustrating the utilization of a 2-axis galvano mirror within the optical pathway to enable spiral scanning of a predefined circular area. (**C**) A table detailing the exposure times and the variations in the angle of the galvano mirror corresponding to different measurement areas. (**D**) A bright field microscopy image displaying the morphological features of induced ANs at 40 days post-induction. The scale bar represents 50 µm. The area subjected to PRESS measurement is demarcated by a red line. (**E**) The average Raman spectra derived from ANs using the PRESS, with SDs depicted as shaded areas. These data aggregated measurements from 30 ganglia (*n* = 30). (**B**,**C**) Figures were generated using BioRender Inc. (Toronto, ON, Canada).

Next, we examined whether the collective output of the neuronal population could be assessed. Autonomic neurons (ANs), differentiated from hiPSCs using the methodology outlined by Takayama et al. [12,13], exhibited a ganglion-like morphology (Figure 2A). The induction of ANs involved their differentiation into neural crest cells using dual SMAD inhibitors combined with CHIR99021, which activated WNT signaling. Furthermore, differentiation into the autonomic nervous system was promoted using a combination of WNT signaling inhibitor (IWR1), SHH signaling inhibitor (SANT1), and recombinant BMP4 (Figure S2A). The neurons induced using this method expressed peripheral nerve markers such as peripherin (PRPH) and autonomic nervous system markers such as PHOX2B, tyrosine hydroxylase (TH), and choline acetyltransferase (CHAT) (Figure S2B). The PRESS was used to obtain the Raman spectra from the ganglia. The dimensions of the measurement area could be tailored to the specimen by setting a specific circular region via the PC interface. For a single cell, as shown in Figure 1, the diameter setting ranges from 5 to 12 μm, while for ganglia, it extends from 40 to 70 μm. It is imperative to adjust the exposure time based on the measured area, which is determined by the oscillation angle of the galvano mirror. Considering the optical trajectory from the galvano mirror to the specimen, the maximum angular variation of the mirror is estimated to be 0.0082, 0.0328, and 0.0574 mrad for circular regions measuring 10, 40, and 70 μm in diameter, respectively. Utilizing this apparatus, a 40 μm diameter circular region was delineated within a ganglion to acquire Raman spectra, setting the exposure time at 5 s (Figure 2D,E). The processed spectral data, after smoothing and background correction, revealed distinct peaks indicative of nucleic acids (748 and 1337 cm^−1^), proteins (1000, 1126, 1337, 1447, 1585, and 1660 cm^−1^), and lipids (1126, 1301, 1447, 1660, and 2930 cm^−1^), along with mitochondria-dependent peaks attributable to cytochrome c (1585 cm^−1^). A comprehensive breakdown of these molecular constituents is shown in Table 1. 

To evaluate the utility of spectra obtained from expanded measurement areas for cell classification, Raman spectra from hiPSCs and ANs were acquired under identical conditions (measurement area: 40 μm diameter circle; exposure time: 5 s) to evaluate their potential for distinguishing between these cell types (Figure S3). Thirty locations were randomly selected from both the hiPSCs and AN ganglion, from which the Raman spectra were obtained (Figure S2A). Initially, there were no visually discernible characteristic peaks between the two cell types (Figure S2B, hiPSCs: black line; ANs: red line). To identify the distinctive peaks and variations in the peak patterns, PCA was employed to process the data. The PCA results demonstrated that hiPSCs and ANs were distinctly separated along the PC1 axis, indicating that the classification was feasible (Figure S3C). Further analysis of the load vectors along the PC1 axis highlighted the significance of the wavenumbers 677, 998, 1126, 1435, and 2843–2891 cm^−1^, with 667 cm^−1^ associated with nucleic acids, 998 cm^−1^ associated with proteins, and 1126, 1435, and 2843–2891 cm^−1^ associated with lipids (Figure S3D). The specific contributions of each peak are listed in Table S1. To assess the reliability of the PCA-based classification model, we implemented a k-fold cross-validation process. The data were split into training and test sets, with PCA applied to the training set and subsequently transforming both sets. A classifier was trained to the transformed training set and evaluated on the test set. This process was repeated five times, yielding an average accuracy of one. These results underscore the robustness and high predictive capability of our PCA-based classification approach. Consequently, the use of the PRESS with an expanded measurement area successfully demonstrated that the Raman spectra derived from neuronal populations are suitable for cell classification.

### 2.3. Evaluation of Nicotine Responsiveness Using Calcium Imaging

**Figure 3:**
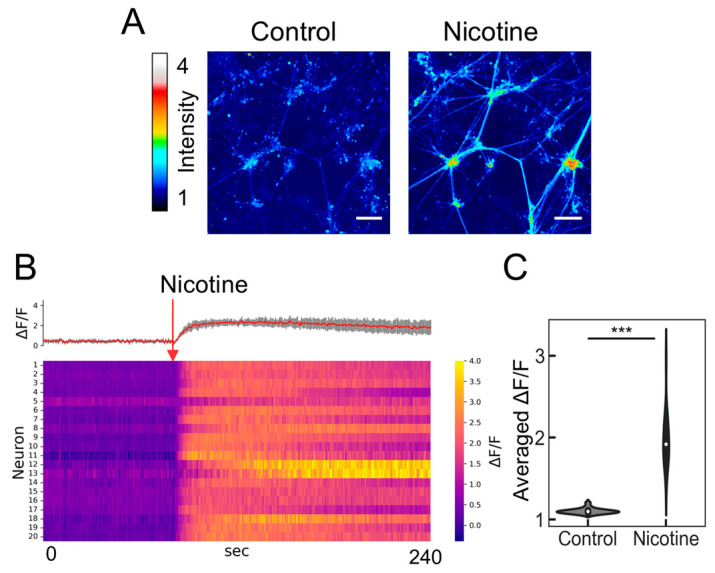
Calcium imaging of induced neurons responding to nicotine. (**A**) Color-coded representation of fluorescence intensity changes indicating calcium influx under control conditions (baseline prior to nicotine stimulation). The scale bar represents 100 µm. (**B**) Heatmap of calcium signaling for 20 neurons exposed to 5 µM nicotine, featuring average calcium transient traces (red) with SDs depicted as shaded gray areas. The red arrow marks the point of nicotine stimulation. (**C**) Violin plot depicting the relative change in average fluorescence intensity (ΔF/F) following the addition of the reagent (ΔF) compared to the baseline (F) (*n* = 20; data were analyzed using the Student’s *t*-test; ***: *p* < 0.001 vs. the control (nicotine-free solution)).

Before evaluating the neuronal population output with the PRESS, we investigated the agonist responsiveness of the induced ANs using an established method of calcium imaging. Given the expression of nicotinic acetylcholine receptors (nAChRs) in ANs [31], nicotine was used as the nAChR agonist (Figure 3A). We randomly selected 20 cell locations and measured the changes in fluorescence intensity upon nicotine addition using heat mapping (Figure 3B). All examined cells exhibited an increase in fluorescence intensity, with the change ratio significantly diverging from that observed in samples treated with a nicotine-free solution (Figure 3C; *p* = 8.78 × 10^−10^, *n* = 20). Further validation was provided by microelectrode array assays, which verified alterations in extracellular membrane potential following nicotine exposure [12,13,32]. Consequently, ANs derived from hiPSCs are functional and capable of reacting to nicotine by initiating neuronal activity via calcium influx.

### 2.4. Assessment of Nicotine Responsiveness of ANs Utilizing the PRESS

**Figure 4:**
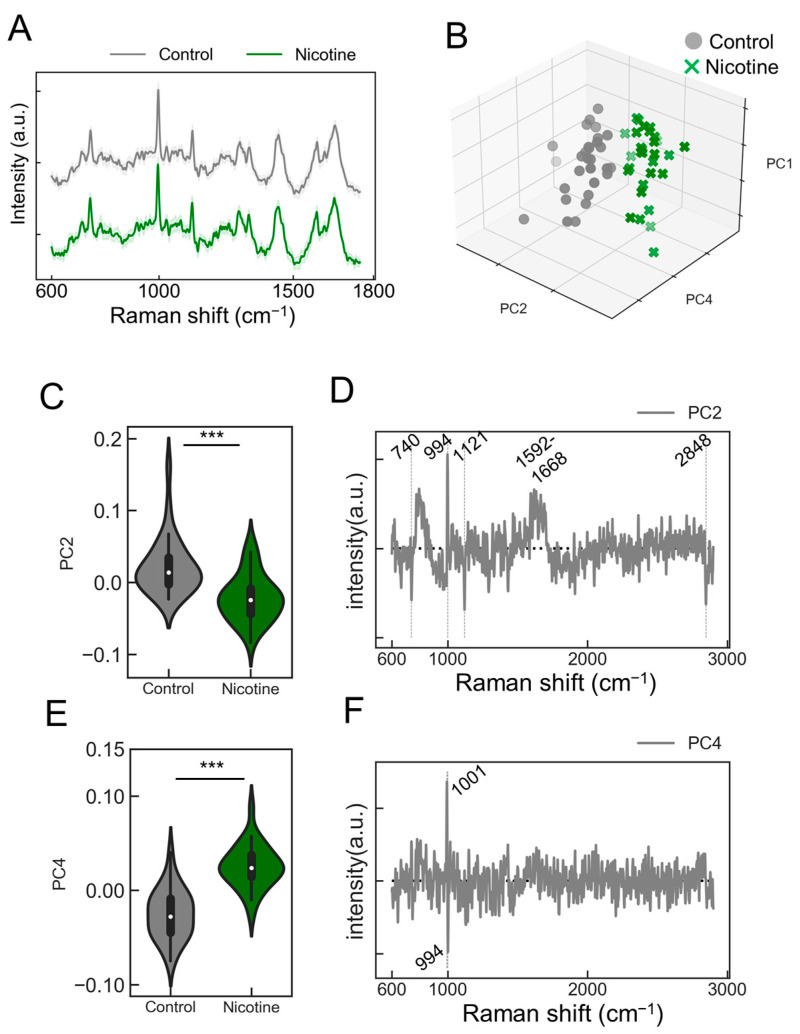
Evaluating the responsiveness of ANs to nicotine stimulation. (**A**) Average Raman spectra obtained from ANs stimulated with nicotine (green) or control buffer (gray). SDs are shown by shaded areas. These data are the result of measurements taken from 30 ganglia (*n* = 30). (**B**) A 3D plot representing the distribution of ANs data points along the PC1, PC2, and PC4 axes from the PCA (control samples: black circles; nicotine-stimulated samples: green crosses [x]). (**C**,**E**) Violin plots comparing values for samples treated with nicotine or control against the PC2 or PC4 axes, respectively (*n* = 30; data were analyzed using the Student’s *t*-test; ***: *p* < 0.001 vs. the control). (**D**,**F**) A display of the loading vectors derived from the PCA for PC2 and PC4.

We explored the capability of the PRESS to detect agonist responsiveness in neurons. Induced neurons stimulated with nicotine were analyzed to collect Raman spectra (Figure 4A, control: gray line; nicotine: green line). The absence of visually altered peaks in the spectral data prompted us to employ PCA for dimensionality reduction and classification (Figure S4). The contributions of the five principal components (PC1–5) were 18.3, 6.0, 5.1, 4.3, and 3.1%, respectively. The PCA results depicted along the PC1, PC2, and PC4 axes are presented in Figure 4B. This analysis revealed that neurons stimulated with nicotine displayed a distribution distinct from those treated with the control buffer, indicating a unique state of neuronal activation (Figure 4B). To further validate the PCA-based classification model, k-fold cross-validation was performed, resulting in a classification accuracy of 0.98. Significant changes in the PC2 and PC4 axes were observed with the addition of nicotine (Figure 4C,E; PC2: *p* = 1.51 × 10^−5^; PC4: *p* = 6.69 × 10^−11^; *n* = 30). The analysis of the load vectors for the PC2 and PC4 axes identified characteristic wavenumbers at 740, 994, 1001, 1121, 1592–1668, and 2848 cm^−1^ (Figure 4D,F). These wavenumbers correspond to various molecular vibrations: 740 cm^−1^ to the ring breathing mode of purine bases in nucleic acids, 994 cm^−1^ to the symmetric stretching vibration of O-P-O, 1001 cm^−1^ to phenylalanine’s breathing vibration, 1121 cm^−1^ to the C-O single bond stretching vibrations in ribose, 1592–1668 cm^−1^ to the amide I band related to peptide bonds in proteins and C=C stretching vibrations, and 2848 cm^−1^ to the symmetric stretching vibrations of methylene groups in lipids. Detailed molecular information is provided in Table 2. Notably, upon nicotine stimulation, increases in scattered light intensity were confirmed at 740, 994, 1121, and 2848 cm^−1^ (Figure S5), potentially serving as Raman markers for AN responses to nicotine. 

### 2.5. The Detection of Intracellular Calcium Ion Fluctuations

**Figure 5:**
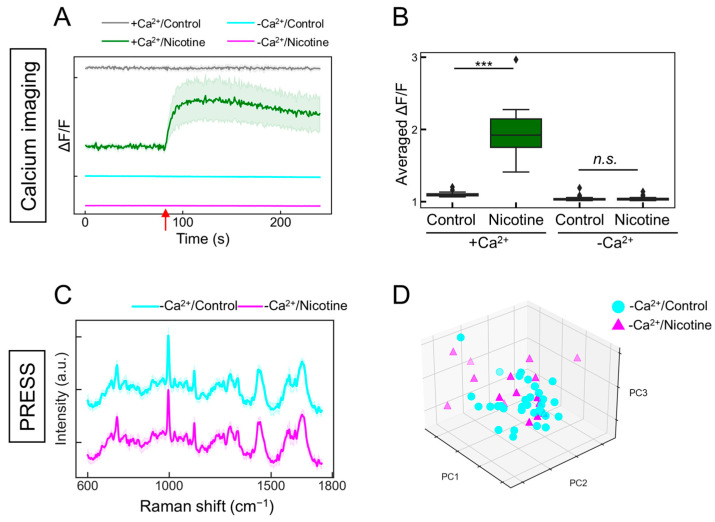
Evaluating the responsiveness of ANs to nicotine stimulation in the absence of calcium ions. (**A**) Time-dependent alterations in intracellular calcium concentrations in induced ANs responding to 5 µM nicotine administration, both with and without calcium ions. In the presence of calcium (+Ca^2+^), control samples are shown in gray and nicotine-stimulated samples are depicted in green. In the absence of calcium (−Ca^2+^), control samples are shown in cyan and nicotine-stimulated samples are depicted in magenta. SDs are shown by shaded areas (*n* = 20). The red arrow signifies the moment of nicotine stimulation (80 s). (**B**) Boxplot depicting the change ratio in average fluorescence intensity (ΔF/F) following the addition of the ligand (ΔF) compared to before ligand addition (F) (*n* = 20; data were analyzed using the Student’s *t*-test; ***: *p* < 0.001; n.s.: not significant vs. the control). (**C**) Average Raman spectra obtained from ANs stimulated with 5 µM nicotine (magenta) or control buffer (cyan) in the absence of calcium ions, with SDs denoted by the shaded areas. Data are based on measurements from more than 16 locations (control: *n* = 32; nicotine: *n* = 16). (**D**) A 3D plot of the AN data points along the PCA’s PC1, PC2, and PC3 axes (control samples: cyan circles; nicotine-stimulated samples: magenta triangles).

The changes observed in Figure 4 prompted a further investigation into whether they accurately reflected neuronal activity. nAChR is a ligand-gated ion channel, and the respective binding of nicotine induces a structural alteration that opens the channel, facilitating the influx of sodium and calcium ions, thereby altering the intracellular potential [36]. It was hypothesized that in the absence of extracellular calcium ions, the addition of addition would not elicit changes in neuronal activity. Indeed, calcium imaging demonstrated that under calcium-depleted conditions, the nicotine-induced increase in intracellular calcium concentration was negated, resulting in the absence of observable neuronal activity (Figure 5A,B; with calcium/control: gray line; with calcium/nicotine: green line; without calcium/control: cyan line; without calcium/nicotine: magenta line). Similarly, when evaluating the neuronal responsiveness to nicotine under calcium-free conditions using the PRESS, the characteristic peaks were indiscernible in the spectral data (Figure 5C; without calcium/control: cyan line; without calcium/nicotine: magenta line). The subsequent PCA for dimensionality reduction and classification did not distinctly separate the neuronal changes induced by nicotine across the PC1–5 axes (Figure 5D and Figure S6; PC1: *p* = 0.201, PC2: *p* = 0.993, PC3: *p* = 0.064, PC4: *p* = 0.670, and PC5: *p* = 0.389 vs. 0 µM, *n* = 16–32). The contributions of PC1–5 were 20.3, 12.7, 6.5, 5.9, and 4.1%, respectively. The accuracy value by k-fold cross-validation was 0.65, indicating that they could not be classified. Therefore, these findings suggest that the PRESS can capture the neuronal activity associated with alterations in calcium dynamics. 

### 2.6. Dose-Dependent Neuronal Dynamics Detected by the PRESS

**Figure 6:**
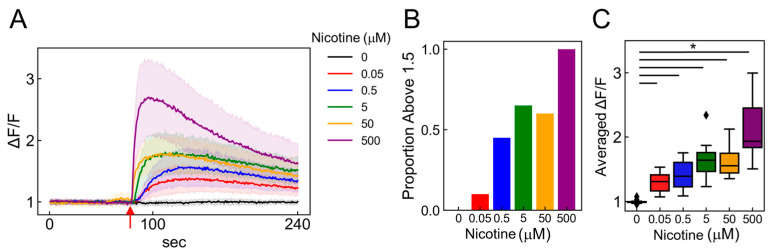
Detecting nicotine concentration-dependent changes using calcium imaging. (**A**) Time-dependent intracellular calcium ion flux changes in induced ANs in response to 0–500 µM nicotine applications. The mean of 20 randomly selected cells is depicted as a solid line, with the SDs indicated by shaded areas. Red arrows signal the moment of nicotine stimulation (80 s). (**B**) Bar graph depicting the percentage of cells exhibiting more than a 1.5-fold increase in fluorescence intensity after nicotine stimulation among 20 selected cells. (**C**) Boxplot illustrating the ratio of change (ΔF/F) in average fluorescence intensity post-ligand addition (ΔF) compared to pre-ligand addition (F) (*n* = 20; data were analyzed using a one-way ANOVA followed by Williams’s post-hoc test. *: *p* < 0.05 vs. ligand-free samples (0 µM)). (**A**–**C**) Colors represent different nicotine concentrations: 0 mM (black), 0.05 (red), 0.5 (blue), 5 (green), 50 (orange), and 500 (purple).

**Figure 7:**
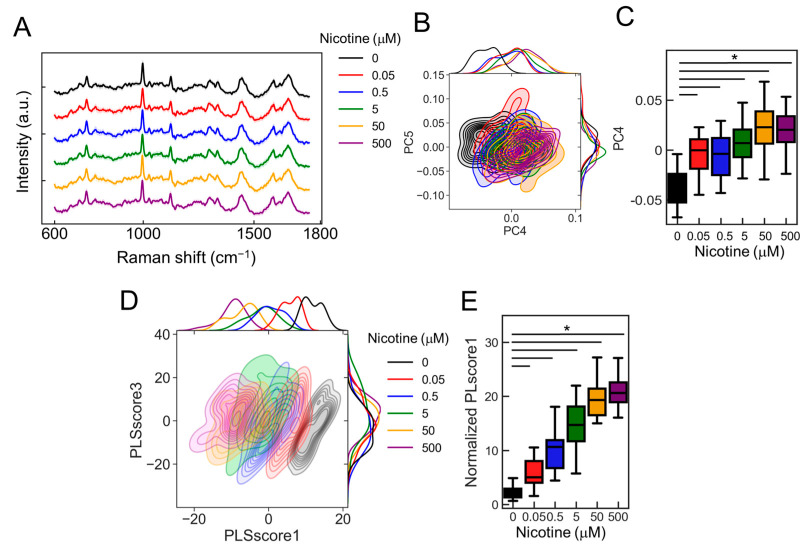
The detection of nicotine concentration-dependent changes using the PRESS. (**A**) Average Raman spectra acquired using the PRESS from ANs stimulated with nicotine concentrations ranging from 0 to 500 µM. SDs are denoted by shaded areas. Data are derived from 30 ganglia. (**B**) A kernel density estimation diagram displaying ANs data points along the PC4 and PC5 axes of the PCA. (**C**) Boxplot illustrating values for each sample plotted against the PC4 axis (*n* = 30; data were analyzed using a one-way ANOVA followed by Williams’s post-hoc test. *: *p* < 0.05 vs. ligand-free samples (0 µM)). (**D**) Kernel density estimation diagram showing ANs data points along the PLScore1 and PLScore3 axes of PLS-DA. (**E**) Boxplot of values for each sample against the PLScore1 axis (*n* = 24; data were analyzed using a one-way ANOVA followed by Williams’s post-hoc test. *: *p* < 0.05 vs. ligand-free samples (0 µM)). (**A**–**E**) Colors represent different nicotine concentrations: 0 mM (black), 0.05 (red), 0.5 (blue), 5 (green), 50 (orange), and 500 (purple).

To estimate classification accuracy, we examined the responses of ANs to various nicotine concentrations. Using calcium imaging to stimulate neurons with nicotine concentrations of 0, 0.05, 0.5, 5, 50, and 500 µM (Figure 6A; 0 mM: black, 0.05: red, 0.5: blue, 5: green, 50: orange, and 500: purple), we observed that both the number of responding cells and the fluorescence intensity significantly increased in a concentration-dependent manner (Figure 6B,C; *p* < 2.00 × 10^−16^, vs. 0 µM, *n* = 20). We explored whether these concentration-dependent changes could be detected using the PRESS. Accordingly, we collected 30 random Raman spectra from the AN ganglia region exposed to nicotine concentrations of 0–500 µM (Figure 7A). The PCA applied to the spectral data revealed distribution changes along the PC4 axis that depended on the nicotine concentration. The contributions of PC1–5 were 14.3, 6.5, 4.1, 2.7, and 2.8%, respectively (Figure 7B). Furthermore, by analyzing values of PC4, states significantly distinct from the control condition (0 µM) were identified at nicotine concentrations exceeding 0.05 µM (Figure 7C and Figure S7A; *p* < 2.00 × 10^−16^, vs. 0 µM, *n* = 30).

To further refine the classification accuracy, we employed PLS-DA, a supervised machine learning technique. This approach provided a more visually distinct representation of concentration-dependent cellular changes than PCA (Figure 7D). The PLscore1, derived from the identified features, showed a marked increase with rising nicotine concentrations compared to the control (0 µM) (Figure 7E and Figure S7B; *p* < 2.00 × 10^−16^, vs. 0 µM, *n* = 24). Moreover, upon evaluating the classification accuracy of each concentration against the control (0 µM) using AUC values, we achieved an AUC of 0.95 for 0.05 µM, 0.93 for 0.5 µM, and 1 for concentrations of 5 µM or higher. Thus, these results indicate that the classification accuracy improves with increasing nicotine concentration, with concentrations of 5 µM and above particularly effective in activating the neurons. 

## 3. Discussion

In this study, we hypothesized that the broad measurement capability and rapid functioning of the PRESS lends itself to the assessment of neuronal output. To test this hypothesis, we evaluated individual neuronal activity using glutamatergic neurons and the neuronal population output in the autonomic ganglia. The results showed that the PRESS could accurately assess not only the glutamatergic responsiveness of individual neurons but also the nicotinic responsiveness of the ganglion. A spectrum derived from a ganglion is the average spectrum derived from multiple cells present in the measurement region. This averaged spectrum showed no decrease in the signal-to-noise ratio compared with the spectrum derived from a single neuron (Figure 1C and Figure 4A). This was expected because collecting signals from multiple cells simultaneously results in the stacking of signals from each cell, which increases the overall signal intensity and averages out the random noise during the measurement, thereby improving the signal-to-noise ratio. However, there are concerns that the larger the measurement area, the longer it takes to acquire one spectrum, the more frequently cosmic rays are detected, and specific information on individual cells will be lost. In summary, these results indicated that the PRESS can be evaluated for individual neurons or neuronal populations by adjusting the measurement area. The PRESS is also expected to be applicable for the evaluation of various neuronal activities, including glutamatergic and nicotinic responses.

When the PCA was performed using the spectra obtained from hiPSCs and ANs, a clear discrimination between hiPSCs and ANs was achieved along the PC1 axis, revealing five characteristic Raman spectral peaks. Notably, the detected lipid-related peak intensities (1435 and 2843–2891 cm^−1^) were considerably higher in ANs than in hiPSCs. Given that lipids are crucial components of cell membranes that influence cellular physical properties, signal transduction, and intercellular interactions, these Raman spectral data potentially shed light on changes in neuronal plasma membrane composition and activities, such as lipid metabolism. These observations are consistent with previous studies that have documented an increase in lipid components during the differentiation of hiPSCs into neural stem cells and neurons, corroborating our findings [37]. However, the peaks identified at 677, 998, and 1126 cm^−1^ have not been previously reported in autonomic neurons, suggesting that these peaks have the potential to serve as specific markers for ANs. This finding warrants further investigation through a comparative analysis of a broad spectrum of neuronal types, including central, motor, and sensory neurons. This necessitates further research in this field.

The nAChR is a ligand-gated ion channel. When the nicotine agonist binds to the nAChR, the ion channel opens, and sodium and calcium ions flow into the neuron, triggering an action potential. Neurotransmitter release occurs only a few milliseconds after the activation of the nAChR [36], followed by short-term effects lasting from a few seconds to several minutes [38]. nAChR activation can have several hour-long effects, including modulation of neurotransmitter release, intracellular signaling pathways such as PI3-Akt, and regulation of gene expression, particularly of cAMP-responsive element-binding protein (CREB), which is known to have long-term effects [39,40]. Raman spectra also showed fluctuations in the peaks contributing to nucleic acids, protein composition, and fatty acids, which may capture long-term intracellular molecular changes.

Thus, the PRESS has been shown to be capable of capturing a comprehensive array of intracellular signal changes. However, one challenge with Raman spectroscopy is its limitation in identifying specific molecular signals in spectral data. In 2018, Kobayashi et al. proposed a methodology for inferring variations in the transcriptome using Raman spectra by treating both Raman spectral and transcriptome data as vectors to identify correlations between them [41,42]. Despite this advancement, the direct identification of specific molecules or networks from Raman spectra is yet to be achieved. The continued development of sophisticated analytical techniques that can be integrated with omics remains a pivotal focus.

The temporal resolution of the PRESS, which is only a few seconds, limits its ability to evaluate responses immediately after stimulation. However, unlike Ca^2+^ imaging, which requires fluorescent probes, the PRESS allows direct measurement in the culture medium without labeling. This capability makes it suitable for observing long-term intracellular molecular changes in neurons, such as those occurring during differentiation, as well as for the direct evaluation of biological tissues during surgical procedures. Moreover, leveraging the spectra of cellular changes induced by various stimuli using advanced machine learning algorithms can enhance the accuracy of predicting neuron activation pathways. To evaluate the responses immediately after stimulation, future enhancements in temporal resolution might involve employing technologies such as Coherent Anti-Stokes Raman Scattering (CARS) [43] and Surface-Enhanced Raman Scattering (SERS) [44,45,46], which can quickly capture highly sensitive Raman signals, along with improvements in scanning systems.

Various techniques have been developed to evaluate the neuronal activity in vivo and in vitro. These include calcium imaging, microelectrode arrays (MEAs), patch clamps, antibody staining for cFOS proteins, mass spectrometry imaging (MSI), magnetoencephalography (MEG), functional magnetic resonance imaging (fMRI), and positron emission tomography (PET). Although calcium imaging and patch clamp methods allow single-cell evaluations, they are invasive, require labeling, and are restricted to the assessment of specific molecules. The MEA approach, which is suitable for neuronal network assessments, evaluates only the neurons in contact with the electrodes and may not capture specific cells or intracellular molecular information. PET, which targets neuronal populations, employs radioisotopes, thus limiting detectable molecules and complicating the comprehensive understanding of entire cell population functions. Conversely, our study demonstrated that the PRESS can nondestructively and label-free detect not only single-cell outputs but also neuronal population outputs, capturing both activity changes and concentration-dependent alterations. 

This study illustrates the utility of Raman spectroscopy for evaluating neuronal activity and drug responsiveness, with promising applications in drug screening and quality control of cellular products essential for regenerative medicine. Furthermore, this technology is not limited to the nervous system and can be extended to various cell types and disease models, offering broad applicability across medicine, biology, and pharmacology.

## 4. Materials and Methods

### 4.1. Cells

hiPSCs (201B7 line, female; RIKEN BioResource Research Center, Tsukuba, Japan) were maintained in mTeSR1-cGMP feeder-free maintenance medium (Stemcell Technologies, Vancouver, BC, Canada) on Laminin511-E8 (iMatrix511; Nippi, Tokyo, Japan)-coated culture plates. The culture medium was changed every day. When the hiPSCs reached confluence, the colonies were digested into single cells using Accutase (Thermo Fisher Scientific, Waltham, MA, USA), and the cells were passaged or used for Raman spectroscopy. 

### 4.2. Glutamatergic Neuron Induction

The differentiation of iPSCs into glutamatergic neurons was enhanced using an optimized protocol based on previously reported methods [10,11,47]. Initially, iPSCs were seeded into 6-well plates coated with 2-methacryloyloxyethyl phosphorylcholine (MPC) (Lipidure CM5206E; NOF, Tokyo, Japan) at a density of 1–2 × 10^5^ cells cm^−2^. These plates were then placed on a rotary shaker set at 95 rpm (OS-762RC, Optime, Tokyo, Japan) and maintained in mTeSR1 medium supplemented with 10 μM Y-27632 (FUJIFILM Wako Pure Chemical Industries, Osaka, Japan) for 2–3 days to form embryonic bodies (EBs).

Subsequently, the EBs underwent a series of medium changes to promote neuronal differentiation. From day 0 to day 5, EBs were cultured in knockout serum replacement (KSR) medium, consisting of DMEM-F12 (FUJIFILM Wako Pure Chemical Industries), 20% KSR (Thermo Fisher Scientific), 1% non-essential amino acids (NEAA), 1% monothioglycerol, and 1% penicillin/streptomycin (P/S), supplemented with 2 μM dorsomorphin (DM; Sigma-Aldrich, St. Louis, MO, USA), 10 μM SB431542 (SB; Sigma-Aldrich), and 10 ng mL^−1^ basic fibroblast growth factor (bFGF). This medium was adjusted on days 5–7 and 7–9 to KSR/N2 medium ratios of 3:1 and 1:1, respectively, containing the same supplements. From days 9–13, the ratio was adjusted to 1:3. The N2 medium consisted of DMEM-F12, 1% N2 supplement (FUJIFILM Wako Pure Chemical Industries), 1% NEAA, and 1% P/S.

On day 13, the differentiated EBs were dissociated using TrypLE Express (Thermo Fisher Scientific), and the resulting cells were plated on dishes coated with poly-L-ornithine (PLO, Sigma-Aldrich) and iMatrix511 (Nippi) in glutamatergic neuronal differentiation (GND) medium. The GND medium was composed of DMEM-F12, neurobasal medium (Thermo Fisher Scientific), 0.5% N2 supplement, 1% B-27 supplement minus vitamin A (Thermo Fisher Scientific), 10 μM forskolin (FSK), 50 μg mL^−1^ ascorbic acid (AA), 10 ng mL^−1^ recombinant human brain-derived neurotrophic factor (BDNF), 10 ng mL^−1^ recombinant human glial cell-derived neurotrophic factor (GDNF), and 10 μM N-[N-(3,5-Difluorophenacetyl)-L-alanyl]-S-phenylglycine t-butyl ester (DAPT), all sourced from FUJIFILM Wako Pure Chemical Industries. Cells were plated at densities between 2.5 and 5 × 10^5^ cells cm^−2^, and the medium was refreshed twice weekly.

### 4.3. AN Induction

The method of inducing the differentiation of human iPSCs into autonomic neurons followed previous reports [12,13,32]. As a similar initial step in glutamatergic neurons, iPSCs are seeded in 6-well plates coated with MPC at a density of 1–2 × 10^5^ cells cm^−2^, and EBs are formed in mTeSR1 medium with 10 μM of Y-27632 for the first 2–3 days.

The subsequent stages involve modifications tailored to autonomic neuron characteristics. From day 0 to day 2, EBs were cultured in KSR medium enriched with 2 µM DM, 10 µM SB431542, and 10 ng mL^−1^ bFGF. The composition of the medium was altered over subsequent days to support the development of specific autonomic neurons. From days 2 to 5, the culture medium transitioned to KSR containing 3 µM CHIR99021 (CHIR; Cayman Chemical, Ann Arbor, MI, USA), 20 µM SB431542, and 10 ng mL^−1^ bFGF. On days 5 to 7, EBs were nurtured in a KSR/N2 medium blend (3:1) with continued supplementation of 3 µM CHIR99021 and 10 ng mL^−1^ bFGF. The ratio changed to 1:1 from days 7 to 9, introducing 10 µM IWR1 (Sigma-Aldrich), 250 nM SANT1 (Sigma-Aldrich), 25 ng mL^−1^ recombinant human bone morphogenetic protein 4 (BMP4; FUJIFILM Wako Pure Chemical Industries), and 10 ng mL^−1^ bFGF. For the final stage, from days 9 to 13, the culture medium consisted of a KSR/N2 blend (1:3) containing the same growth factors, with a medium change on day 12.

On day 13, the differentiated EBs were dissociated using TrypLE Express and seeded onto dishes coated with PLO and laminin (Sigma-Aldrich) in neuronal differentiation (ND) medium. The ND medium included the N2 medium, 10 μM FSK, 50 μg mL^−1^ AA, 10 ng mL^−1^ BDNF, 10 ng mL^−1^ GDNF, 10 ng mL^−1^ recombinant human nerve growth factor β (NGFβ), and 10 ng mL^−1^ recombinant human neurotrophin 3 (NT-3). The cells were plated at a density of 2.5–5 × 10^5^ cells cm^−2^, with medium changes twice weekly to promote maturation into ANs suitable for subsequent analyses, including Raman spectroscopy in phenol red-free DMEM/F12 and mTeSR1 media.

### 4.4. Calcium Imaging

The samples were incubated with 10 mM Fluo-8 AM, a calcium indicator (AAT Bioquest, Pleasanton, CA, USA), in the ND medium at 37 °C for 30 min. Next, the ND medium containing Fluo-8 AM was replaced with Ringer’s solution (148 mM NaCl, 2.8 mM KCl, 2 mM CaCl_2_, 1 mM MgCl_2_, 10 mM HEPES, and 10 mM glucose; pH 7.4). As the agonist, 5 μM nicotine (Sigma-Aldrich) or 10 μM glutamate (in Ringer’s solution; FUJIFILM Wako Pure Chemical Industries) was used. The sample was placed on the stage of an inverted microscope (IX71; Olympus, Tokyo, Japan), and fluorescence was detected using a confocal spinning disk confocal laser scanning unit (CSU-W1; Yokogawa Electric, Tokyo, Japan) and LED light (X-Cite 120LED; OPTO SCIENCE, Tokyo, Japan). For drug application, 0.05–500 μM nicotine was added to Ringer’s solution during fluorescent observation. A frame rate of 0.8 s^−1^ was used. The recorded fluorescence signals were analyzed using ImageJ (1.54g) software [48] (National Institutes of Health, Bethesda, MD, USA; available at http://imagej.nih.gov/ij/ (accessed on 1 July 2024)).

### 4.5. The PRESS

The Raman spectra of the cells were obtained using a custom-built Raman microscope based on a Raman system (Confocal Raman Spectrometer STR series; AIRIX Corp., Tokyo, Japan). The details were presented in a previous report [9]. The Raman system consisted of a Nikon Ti2-U microscope (Nikon, Tokyo, Japan). A continuous-wave Diode-Pumped Solid-State (DPSS) green 532 nm laser (DL 532-100, maximum power 120 mW) coupled with an optical fiber was used for excitation. A Lambda 40× oil C/NA 1.3 objective lens (Nikon, CFI Super Fluor 40× Oil) was used at a power of approximately 40 mW in the sample stage, producing a laser spot size of <460 nm. A laser spot size is calculated by the following formula: spot size = (2 × M^2^ × wavelength)/(π × NA). M denotes the beam quality, which was 1.1 in our system; the wavelength was 532 nm; and the NA was 1.3. Consequently, the spot size was expected to be 315 nm. Backscattered Raman signals were dispersed using a spectrometer (STR200-2LC, AIRIX) equipped with a 1200 L mm^−1^ grating and detected using a CCD camera (iVac316, Andor Technology). To maintain cell culture conditions, the microscope was equipped with a warning box (Tokai Hit., Co, Ltd., Shizuoka, Japan) to allow measurements to be made in a 37 °C environment.

To construct the PRESS for wide-area measurements, two biaxial galvano mirrors were integrated into the optical path of the laser (Figure 2B). A 532 nm DPSS laser beam was directed through an optical fiber, neutral density filter (NDF), and bandpass filter (BPF) and then reflected by the mirrors to the galvano mirrors. These mirrors rotate rapidly to project the laser in a swirling pattern onto the sample. The scattered light retraces the laser path, passing through dichroic mirrors and a high-pass filter (HPF) to isolate the Raman-scattering light, which is then focused onto a spectrometer via an optical fiber. This light was detected by a CCD detector, producing a spectrum with a Raman shift (wavenumber, cm^−1^) on the horizontal axis and intensity (a.u.) on the vertical axis, covering a spectral region of 46–3110 cm^−1^. The number of exposures in an area can be changed by controlling the rotational speed of the galvano mirror. The marking speed of the laser was 0.002–2 mm ms^−1^. The area to be irradiated can be specified as a circle (0.5–100 μm diameter). The angular variation in the galvanometer mirror with respect to the measurement area was obtained using the following angular approximation. The deflection angle (*θ*) of the laser beam depends on the feature size of the sample to be measured (in this case, the diameter of a circle (*D*)) and the focal length of the objective lens (*L*). The resulting angular changes were converted into milliradians (mrad).

(1) θ~D/2L

### 4.6. Raman Data Acquisition 

To calibrate the spectrometer, we measured the spectra of Raman shift standards and sulfur before sample measurement. The sulfur spectrum was detected using a point-scan system with an exposure time of 1 s and an NDF of 5%. Five characteristic spectral peaks were detected (50.0, 85.1, 153.8, 219.1, and 473.2 cm^−1^), and calibration was performed with three points (153.8, 219.1, and 473.2 cm^−1^) (ASTM E1840-96 [49]). 

Glutamatergic neurons were measured using Ringer’s solution. The measurement area was set to a 10 μm diameter circle with an exposure time of 3 s and a marking speed of 2 mm ms^−1^. Thirty cells were analyzed.

hiPSCs and ANs were cultured in mTeSR1 (phenol red-free) or N2 medium containing six growth factors (phenol red-free). The measurement area was set to a 40 μm diameter circle with an exposure time of 5 s and a marking speed of 2 mm ms^−1^. For neurons, the ganglion regions with many cell bodies were selected for measurement. Each of the 30 locations was measured.

### 4.7. Raman Spectrum Analysis

Raman spectra were analyzed using Python 3.11.7 (Continuum Analytics, Inc., Newport Beach, CA, USA). The pre-processing of the data was performed as previously reported [50]. Briefly, the spectra were first smoothed using the Savitzky–Golay function (second polynomial order). Subsequently, the baseline was corrected using a polynomial function (order 4) and vector normalized to the 600–2980 cm^−1^ spectral range. 

### 4.8. Machine Learning Classification

To reduce the dimensionality of the Raman spectrum, a PCA of the data was conducted to extract the PC loading vectors and scores. The first five PCs were selected because they accounted for most of the variance in the dataset. To assess the reliability of the PCA-based classification model, we implemented a k-fold cross-validation process. The data were split into training and test sets, with the PCA applied to the training set and subsequently transforming both sets. A classifier, specifically a Support Vector Machine (SVM), was trained on the transformed training set and evaluated on the test set. This process was repeated five times to obtain the average accuracy.

For the supervised learning analysis, we used smoothed, baseline-corrected, and vector-normalized data before dimensionality reduction by PCA. A PLS-DA was performed. For classification accuracy, we performed cross-validation. All spectral data were mixed and randomly separated into 80% training data and 20% testing data. All data were processed using Python. PCA and PLS-DA were performed using the Scikit-learn Python package. 

We comprehensively evaluated the models based on the receiver operating characteristic (ROC) curves and ten-fold cross validation. In the process of generating the ROC curves, the performance parameters for all the models were calculated based on the sensitivity and specificity of each class, as well as the overall accuracy rate, as follows:

Sensitivity = TP/(TP + FN);(2)

Specificity = TN/(TN+ FP);(3)

Accuracy = (TP + TN)/(TP + TN + FP + FN).(4)

where TP, FP, TN, and FN denote the numbers of true positives, false positives, true negatives, and false negatives, respectively. Using these values, we plotted the ROC curves. The X-axis represents the false-positive rate (FPR, 1—specificity), and the Y-axis indicates the true-positive rate (TPR). The AUC was calculated to determine the accuracy of the model. 

Five PLS scores were allocated to each cell in the training and test datasets to extract the five axes contributing to PLS-DA classification. This complicated the evaluation of the sensitivity and accuracy of the binomial classification. Therefore, using the Mahalanobis distance, the five scores were changed to binomial classification values. First, we calculated the central points of the two classes (activated or naïve cells) created using the training data. We then calculated the Mahalanobis distance between the central points of the two classes and the test data and classified them into classes with lower Mahalanobis distance values. Consequently, the value of each test data point from the five PLS-DA scores was changed to a binomial value. Thus, the sensitivity, specificity, and accuracy can be obtained by reducing the dimensionality to five through PLS-DA and classifying the data into two classes based on the Mahalanobis distance.

### 4.9. Reverse Transcription (RT)-qPCR

Total RNA was isolated from the induced cells using a Nucleospin RNA kit (TaKaRa, Shiga, Japan). RNA purity and concentration were determined using a NanoDrop Lite spectrophotometer (Thermo Fisher Scientific). We reverse-transcribed 1000 ng of total RNA into cDNA using a ReverTra Ace qPCR RT kit (TOYOBO, Osaka, Japan). qPCR was performed in a LightCycler^®^ 96 System (F. Hoffmann-La Roche, Ltd., Basel, Switzerland) using the THUNDERBIRD SYBR qPCR mix (TOYOBO) under the following conditions: 95 °C for 600 s, 60 °C for 10 s, and 72 °C for 10 s; 45 cycles of 95 °C for 10 s, 60 °C for 10 s, and 72 °C for 10 s; and melting at 95 °C for 10 s, 65 °C for 60 s, and 97 °C for 1 s. The expression of the genes was normalized to 36B4 expression (housekeeping gene). The primer sequences are listed in Table S2.

### 4.10. Immunochemical Staining

Immunochemical experiments were performed as previously described [32]. Briefly, induced neurons were fixed in 4% paraformaldehyde (FUJIFILM Wako Pure Chemical Industries) for 10 min, permeabilized using 0.1% Triton X-100 (FUJIFILM Wako Pure Chemical Industries) in phosphate-buffered saline (PBS) for 10 min, and blocked with 4% Block Ace (DS Pharma Biomedical, Osaka, Japan) in 0.01% Triton X-100 for 1 h at room temperature (approximately 25 °C). The following primary antibodies were used: mouse anti-microtubule-associated protein (MAP2; 1:1000; Abcam, ab11267, Cambridge, UK), rabbit anti-peripherin (Peripherin; 1:1000; Abcam, ab246502), rabbit anti-vesicular glutamate transporter 1 (VGLUT1; 1:300; Abcam, ab104898), and Hoechst 33342 (H342, DOJINDO LABORATORIES, Kumamoto, Japan). The following secondary antibodies were used: Alexa Fluor 555 (F[ab])2 goat anti-rabbit IgG [H + L] secondary antibody) (Thermo Fisher Scientific, A21430) and Alexa Fluor 488 (F[ab’]2 goat anti-mouse IgG [H + L] secondary antibody) (Thermo Fisher Scientific, A11017).

### 4.11. Statistics and Reproducibility 

All data are expressed as mean ± SD values. Differences between experimental groups were analyzed using the Student’s *t*-test (two groups). Differences between more than two groups were analyzed using the one-way ANOVA, Tukey’s post-hoc test, and Williams’s post-hoc method, which were used for multiple comparisons. Differences with *p* < 0.05 were considered statistically significant. The symbols used are * *p* < 0.05, ** *p* < 0.01, *** *p* < 0.001, and n.s. (not significant, *p* ≥ 0.05). Statistical analysis was performed using Python 3.11.7 or R 4.3.3 (Statistical Computing, Vienna, Austria).

## 5. Patents

A patent application for the contents of this paper was filed on 15 April 2021 (JP 2022-163844, PCT/JP2022/013239).

## Figures and Tables

**Table 1:** The assignment of specific Raman bands to vibrational models and biological molecules related to Figure 2E [14].

Peak (cm^−1^)	Assignment	Reference
748	DNA	[15]
1000	Phenylalanine Bound and free of nicotinamide adenine dinucleotide hydrogen (NADH) Breathing mode in benzene ring	[16,17,18]
1126	C-C stretching in lipid acyl backbone C-N stretching in proteins	[19,20]
1301	Triglycerides (fatty acids) C-H stretching in lipids CH_2_ twisting in lipids	[21,22,23]
1337	Amide III CH_2_ wagging from the glycine backbone and proline side chain A, G ring breathing in DNA bases C-H bending in proteins	[19,24,25]
1447	CH_2_ bending in proteins and lipids	[19,26]
1585	C=C stretching in proteins cytochrome c	[26,27,28]
1660	Amide I stretching in structural proteins C=C stretching in cis lipids and fatty acids	[20,21,23,29]
2930	CH_2_ asymmetric stretching	[30]

**Table 2:** The assignment of specific Raman bands to vibrational models and biological molecules related to Figure 4D,F [14].

Peak (cm^−1^)	Assignment	Reference
**740**	Nucleotide conformation	[19]
**994**	O-P-O symmetric stretching	[33]
**1001**	Phenylalanine Bound and free NADH Breathing mode in the benzene ring	[16,17,18]
**1121**	C-O stretching in ribose	[24]
**1592–1668**	C=C stretching Tryptophan Protein (Amid I) Bound and free NADH	[16,34]
**2848**	CH_2_ and CH_3_ symmetric stretching in lipids	[17,30,35]

## Data Availability

All data supporting the findings of this study are available from the corresponding author upon request.

## Supplementary Materials

The following supporting information can be downloaded at: https://www.mdpi.com/article/10.3390/molecules29133174/s1, Figure S1: Evaluation of individual neurons activity using the Paint Raman Express Spectroscopy System (PRESS); Figure S2: Characterization of autonomic neurons (ANs) derived from hiPSCs; Figure S3: Classification of hiPSCs and ANs using the PRESS; Figure S4: Evaluating the responsiveness of ANs to nicotine stimulation; Figure S5: Raman peaks detected by nicotine stimulation; Figure S6: Evaluating the responsiveness of ANs to nicotine stimulation in the absence of calcium ions; Figure S7: Detection of nicotine concentration-dependent changes using the PRESS; Table S1: The assignment of specific Raman bands to vibrational models and biological molecules related to Figure S3D ([16,17,18,19,23,25,35,51,52,53]); Table S2. The sequences of primers used for quantitative PCR.
