# Supplementary material for: Label-Free Assessment of Neuronal Activity Using Raman Micro-Spectroscopy
Source: Molecules. 2024 Jul 3;29(13):3174. doi: 10.3390/molecules29133174 (PMC11243074; doi:10.3390/molecules29133174)
Supplement: Supplementary file 1 [file molecules-29-03174-s001.zip › molecules-3078475-supplementary.pdf]

Supplementary information

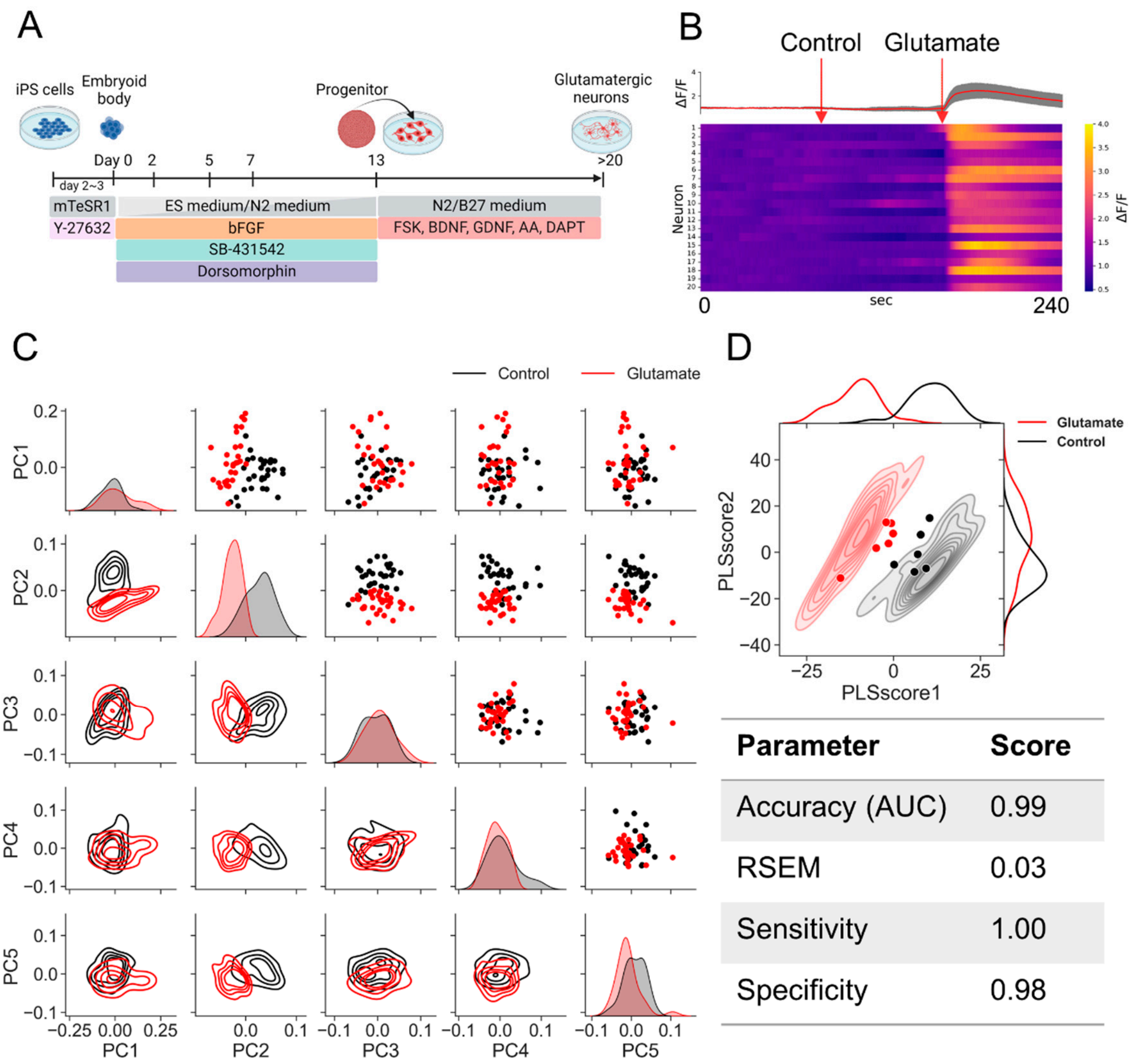

Figure S1. Evaluation of individual neurons activity using the Paint Raman Express Spectroscopy System (PRESS). (A) Schematic of the differentiation process from hiPSCs to glutamatergic neurons, outlining key growth factors and signaling molecules involved: basic fibroblast growth factor (bFGF), Dorsomorphin (DM), Forskolin (FSK), glial cell-derived neurotrophic factor (GDNF), brain-derived neurotrophic factor (BDNF), N-[N-(3,5-Difluorophenacetyl)-L-alanyl]-S-phenylglycine t-butyl ester (DAPT), and ascorbic acid (AA). (The figure was created with Biorender Inc.) (B) Heatmap of calcium signaling for 20 neurons exposed to 10 μM glutamate, featuring average calcium transient traces (red) with SDs depicted as shaded grey areas. The red arrow marks the point of control buffer or glutamate addition. The color scale shows the change value (ΔF) with respect to the mean intensity before the addition of the control buffer (F). (C) Pair plot comparing the first five principal components according to PCA, indicating the separability of classes and the

distributions for classification. The upper panels show scatter plots where each dot represents a single cell. The lower panels show the Kernel density distribution diagram for each cell type. (Control (0 μM) stimulation sample: black, Glutamate (10 μM) stimulation sample: red.) (D) Kernel density estimation diagram showing neurons exposed to 10 μM glutamate data points along the PLScore1 and PLScore2 axes of PLS-DA. The table on the down shows the classification accuracy (area under the curve; AUC), error value (root mean square error; RSEM), sensitivity and specificity calculated by PLS-DA.

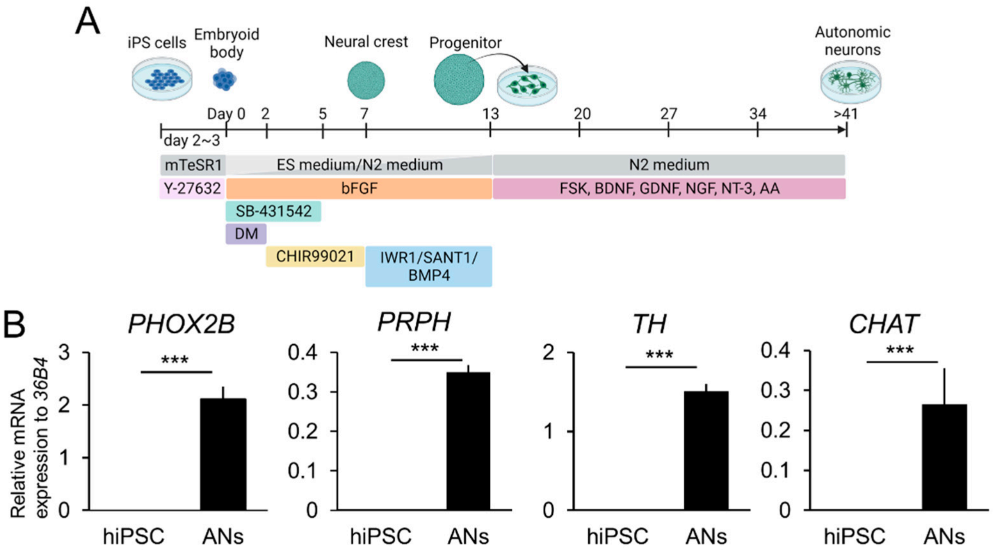

Figure S2. Characterization of autonomic neurons (ANs) derived from hiPSCs (A) Schematic of the differentiation process from hiPSCs to ANs, outlining key growth factors and signaling molecules involved: basic fibroblast growth factor (bFGF), Dorsomorphin (DM), Forskolin (FSK), glial cell-derived neurotrophic factor (GDNF), brain-derived neurotrophic factor (BDNF), nerve growth factor-beta (NGF-β), neurotrophin-3 (NT-3), and ascorbic acid (AA). (The figure was created with Biorender Inc.) (B) Quantitative mRNA expression analysis of paired-like homeobox 2B (PHOX2B), peripherin (PRPH), tyrosine hydroxylase (TH), and choline acetyltransferase (CHAT) relative to the housekeeping gene 36B4 in induced neurons at 40 days post-induction. (*n*=3, data presented as mean ± SDs, data were analyzed using Student's *t*-test, *** $P < 0.001$ vs. hiPSCs).

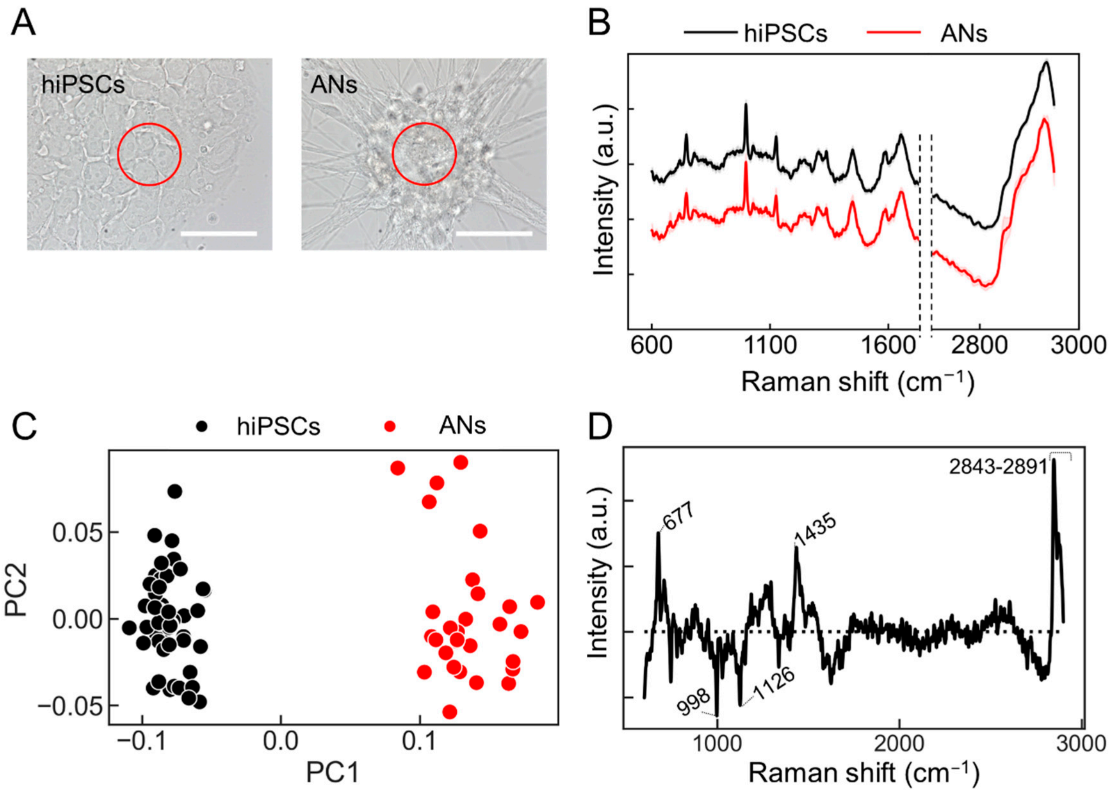

Figure S3. Classification of hiPSCs and ANs using PRESS (A) Bright field microscopy image displaying the morphological features of hiPSCs and ANs at 40 days post-induction. The scale bar represents 50 µm. The area subjected to PRESS measurement is demarcated by a red line. (B) Average Raman spectra derived from hiPSCs (black) and ANs (red) using PRESS, with SDs depicted as shaded areas. The data aggregates measurements from 30 distinct locations. (C) A scatter diagram representing the distribution of hiPSCs (black) and ANs (red) data points along the PC1 and PC2 axes from PCA, illustrating the discernible classification by cell type. (D) Display of the loading vectors derived from PCA for PC1.

Table S1. Assignment of specific Raman bands to vibrational models and biological molecules related to Figure S3D [14].

PC1 Peak (cm^{-1})	Assignment	Reference
677	C-S stretching in cystine (collagen type 1),<br>T, G (DNA/RNA)	[23,25,51]
998	Phenylalanine<br>Breathing mode in benzene ring	[16-18]
1126	C-C stretching in lipid acyl backbone<br>C-N stretching in proteins	[19,25]
1435	CH_2 bending in lipids	[52,53]
2843-2891	CH_2 & CH_3 symmetric stretching in lipid	[35]

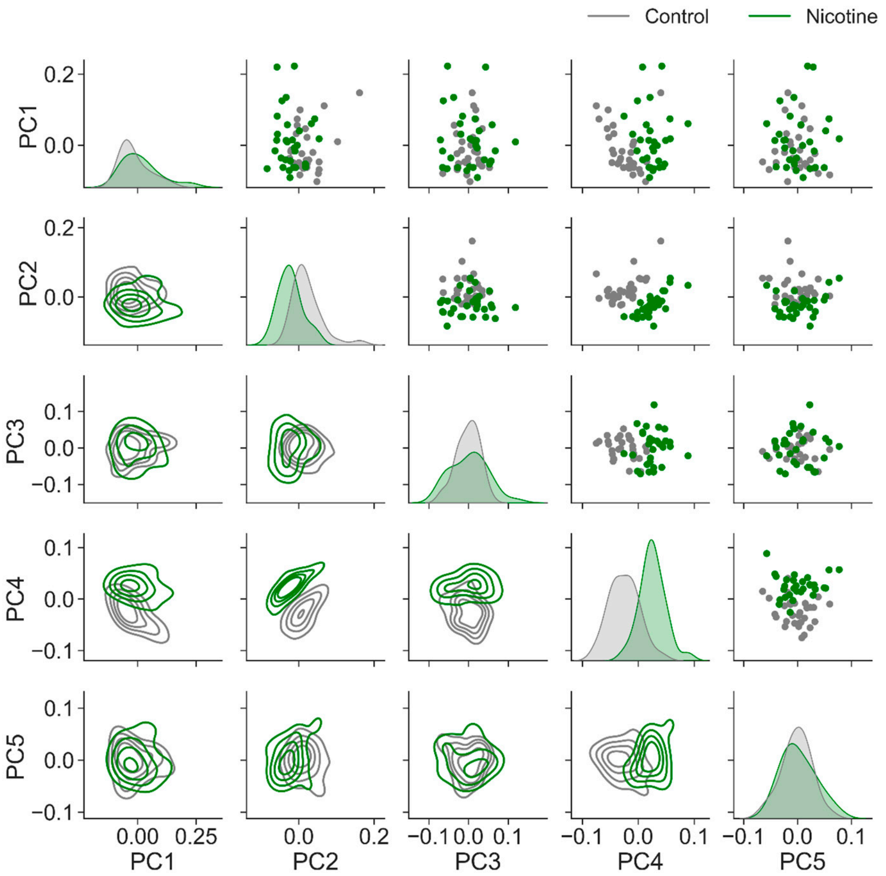

Figure S4. Evaluating the responsiveness of ANs to nicotine stimulation. Pair plot comparing the first five principal components according to PCA, indicating the separability of classes and the distributions for classification. The upper panels show scatter plots where each dot represents a single ganglion. The lower panels show the Kernel density distribution diagram for each cell type. (Control (0 µM) stimulation sample: grey, Nicotine (5 µM) stimulation sample: green).

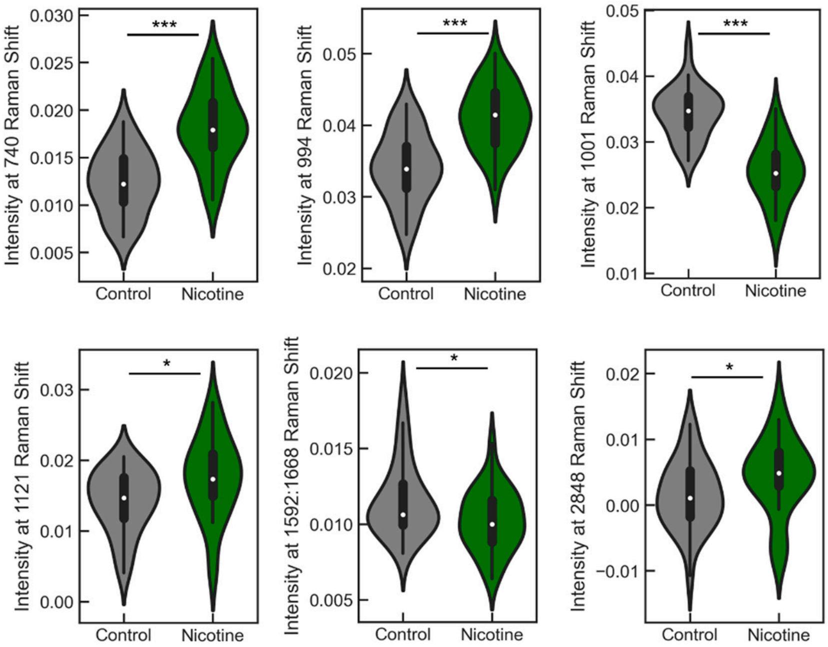

Figure S5. Raman peaks detected by nicotine stimulation. Comparison of scattered light intensities at 740, 994, 1001, 1121, 1592-1668, and 2848 cm^{-1} detected from PC2 and PC4 loading vectors in Figure 4C-F. (n=30, data were analyzed using Student's *t*-test, ***: *P*<0.001, *: *P*<0.05 vs Control)

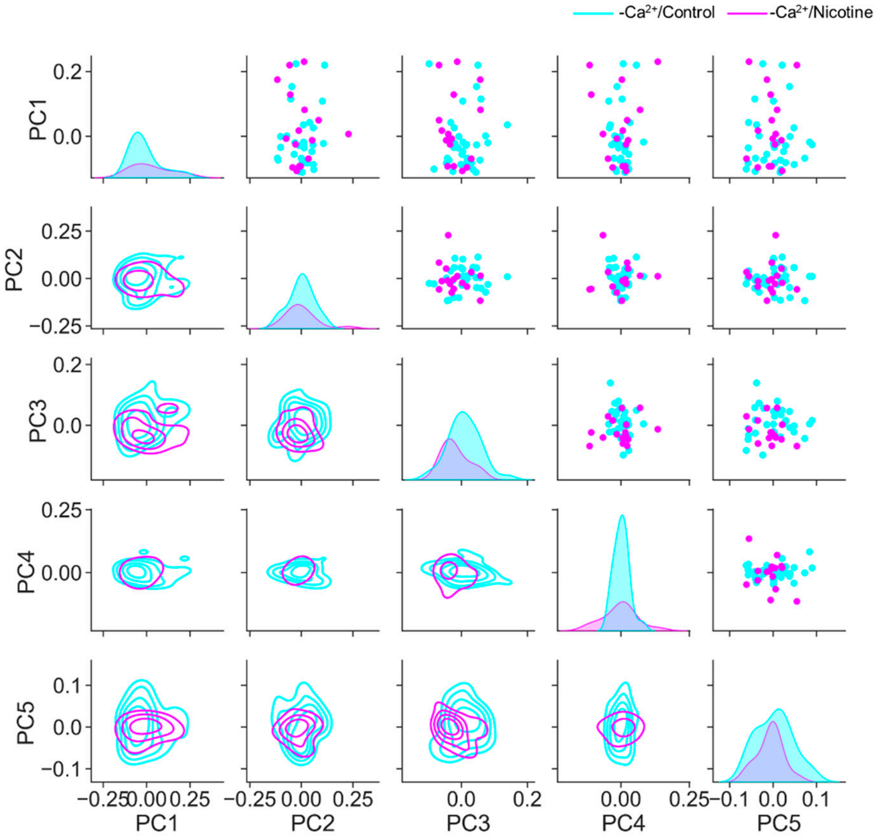

Figure S6. Evaluating the responsiveness of ANs to nicotine stimulation in the absence of calcium ions. Pair plot comparing the first five principal components according to PCA, indicating the separability of classes and the distributions for classification. The upper panels show scatter plots where each dot represents a single ganglion. The lower panels show the Kernel density distribution diagram for each cell type. (Control (0 μM) stimulation sample: cyan, Nicotine (5 μM) stimulation sample: magenta. Both samples were measured under calcium-free condition).

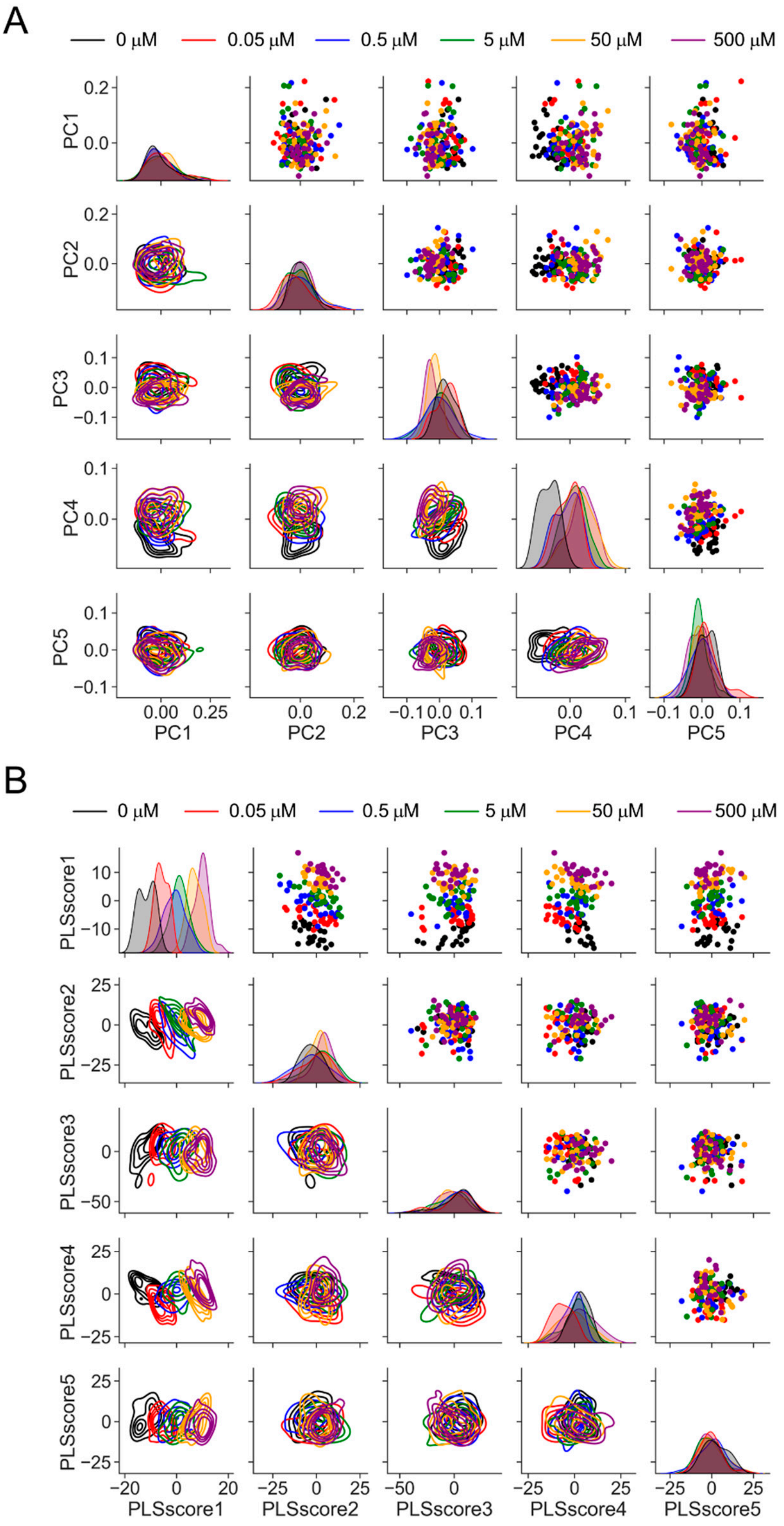

Figure S7. Detection of nicotine concentration-dependent changes using PRESS (A) Pair plot comparing the first five principal components according to PCA, indicating the separability of classes and the distributions for classification. (B) Pair plot comparing the

first five PLS scores according to PLS-DA, showing the separability of classes and the distributions for classification. (A, B) The upper panels show scatter plots where each dot represents a single ganglion. The lower panels show the Kernel density distribution diagram for each sample. Colors represent different nicotine concentrations: 0 μM (black), 0.05 (red), 0.5 (blue), 5 (green), 50 (orange), and 500 (purple).

Table S2. Sequences of primers used for quantitative PCR.

Gene symbol	Forward (5'-3')	Reverse (5'-3')
36B4	AGATGCAGCAGATCCGCA	GTTCTTGCCCATCAGCACC
CHAT	GCCTTCTACAGGCTCCATCG	GGAGTGGCCGATCTGATGTT
PHOX2B	GCTGGCCCTGAAGATCGAC	TCAGACTTTTTGCCCGAGGAG
PRPH	GCCTGGAACTAGAGCGCAAG	CCTCGCACGTTAGACTCTGG
TH	GCGCAGGAAGCTGATTGC	CAATCTCCTCGGCGGTGTAC
